# Beyond accuracy: evaluating the reliability of large language models for medical assessment

**DOI:** 10.3389/frai.2026.1832829

**Published:** 2026-07-08

**Authors:** Hui Zhang, Lihui Qu, Hongbo Bai, Youbang Chen, Ruiying Ji, Zirui Cheng, Chun-tao Yang

**Affiliations:** 1 Office of Academic Affairs, Guangzhou Medical University, Guangzhou, China; 2 Guangzhou Municipal and Guangdong Provincial Key Laboratory of Protein Modification and Disease, Department of Physiology, School of Basic Medical Sciences, Guangzhou Medical University, Guangzhou, China; 3 Zhongshan School of Medicine, Sun Yat-Sen University, Guangzhou, China

**Keywords:** artificial intelligence, assessment automation, cross-run variance, large language models, medical education, reliability, test blueprint

## Abstract

**Background:**

Large language models (LLMs) perform well on medical examinations, but they are almost always evaluated as test-takers, judged on the accuracy of their answers. Far less is known about their reliability as assessment-automation tools, for instance in extracting examination metadata, or about whether single-run, accuracy-based benchmarks can adequately characterize such tools.

**Methods:**

Twelve mainstream LLMs (8 domestic, 4 international) were evaluated on extracting eight metadata fields from a 60-item endocrinology examination, each performing the identical task in three independent runs under realistic web-based conditions. Performance was separated into completion rate, conditional accuracy, and an all-fields-correct task success rate (TSR), with cross-run variance treated as a primary outcome. Conditional accuracy and TSR were compared within models, and domestic versus international models were compared on TSR.

**Results:**

Conditional accuracy was uniformly high, yet TSR was sharply bimodal: five models scored 90% or above and seven below 70%, with none in between. Reliability, not mean accuracy, was decisive. Several models with perfect conditional accuracy collapsed when one or two of their three runs failed entirely, a pattern that single-run evaluation would miss. Failures spanned both model behaviour and platform-level constraints, the latter not removable by prompting. Model origin did not predict performance. On one item, four models independently changed the examiner’s assigned cognitive level to one of their own, a “silent relabeling” with direct implications for item-bank integrity.

**Conclusion:**

For automated assessment metadata extraction, reliability rather than accuracy determines whether an LLM can be deployed, and national origin is not a meaningful predictor. Such tools should be evaluated by repeated runs reporting cross-run variance, and adopted through task-specific piloting within a workflow that reserves human judgement for semantic and normative fields.

## Introduction

The rapid integration of large language models (LLMs) into medical education has, so far, been driven largely by their performance as answerers of questions (Ning et al., 2025). Yet the tasks that most burden educators are not answering questions but managing them. Quality assurance in medical examination requires aligning every test item with its intended content domain, cognitive level, and item type, a process formalized in a test blueprint (two-way specification table). Building such a blueprint demands manual extraction of multiple metadata fields per item (question type, score, cognitive level, cognitive objective, chapter, knowledge points, and difficulty), amounting to 480 field values for a single 60-item examination, work that is time-consuming, expertise-dependent, and difficult to scale. The burden is compounded in many Chinese medical schools by a separation between question-bank platforms, which store tagged items, and examination-management systems (Zhang et al., 2022; Heilongjiang North Zhongtang Zhika Technology Co., Ltd., 2024), which require structured blueprints for assembly; bridging the two means re-extracting and reformatting these fields by hand. Commercial systems that automate this are costly to license and maintain, placing them out of reach for many institutions and motivating the search for low-cost alternatives such as freely available LLMs.

A growing body of research has examined LLMs in medical education, though predominantly along a single line of inquiry. The largest strand evaluates them as test-takers: current models routinely pass national medical licensing examinations, with Chinese-trained models performing comparably to or better than international ones on Chinese-language benchmarks (Wu et al., 2024; Yao et al., 2025; Xiong et al., 2025), at times matching or exceeding clinicians on structured knowledge assessments (Zhang et al., 2025) and improving students’ problem-solving beyond conventional resources (Xavier et al., 2026). A smaller strand examines LLMs as assessment aids (most notably as automated graders), where they show promise but require human oversight for judgement-dependent scoring (Bolgova et al., 2025), echoing frameworks that reserve normative decisions for educators while delegating routine processing to AI (Rareș et al., 2025). A third, recent strand turns from accuracy to reliability, cautioning that even next-generation models perform inconsistently on specialized content and that single-run rankings are unstable, shifting substantially when models are tested repeatedly (Huan and Wang, 2026; Gonzalez et al., 2025; Hajjo et al., 2026). These studies share one unexamined assumption: that a model able to answer questions accurately can therefore carry out a task. But answering well is not the same as performing reliably, and whether LLMs are dependable enough to be deployed, not just accurate enough on a single attempt, has rarely been tested.

This gap has direct practical consequences. Because prior evaluations typically score models on single attempts (Gonzalez et al., 2025) and ask only whether answers are correct, they cannot reveal whether a model would perform the same task dependably on repeated use. That consistency is the property that actually determines deployability. Furthermore, nor have many models been compared head-to-head on an identical task under the conditions an ordinary user faces, leaving institutions to choose among proliferating options with little evidence. Closing this gap requires a shift in how these tools are evaluated: testing them not once but repeatedly, and judging them not by peak accuracy but by the consistency of what they deliver.

We therefore evaluated twelve mainstream LLMs, including eight domestic and four international, on a standardized examination metadata-extraction task, each performing the identical task in three independent runs under realistic web-based conditions. Rather than rank models on accuracy alone, we separated three layers of performance (completion, conditional accuracy, and an all-fields-correct task success rate), and treated cross-run variance as a primary outcome. This reframing proved consequential: model origin did not predict performance, several highly accurate models were undermined by run-to-run failures invisible to single-run testing, and some models went further still, silently overriding the examiner’s own metadata with judgements of their own. By relocating the evaluation of LLMs as assessment tools from accuracy to reliability, this study offers both a cautionary framework and a practical, evidence-based basis for model selection in medical education.

## Methods

### Study design and setting

This comparative evaluation study was conducted at Guangzhou Medical University (Guangzhou, China), with all model runs performed between 25 May and 3 June 2026 (during revision). Using a standardized, task-based protocol, we assessed twelve LLMs on the automated extraction of assessment metadata from a medical-education examination. To quantify not only accuracy but also the run-to-run reliability of each model, every model performed the identical task in three independent runs, yielding 36 runs in total. This repeated-measures design is a deliberate departure from single-run evaluations, which cannot distinguish a consistently capable model from one that succeeds only intermittently.

The test material was drawn from a previously administered examination that is no longer in active use. Before the study, the items were de-identified and processed: all institutional, disciplinary, and examinee-identifying information was removed, and the item stems were rephrased to avoid disclosing the original examination content. This processing preserved each item’s assessed knowledge points, chapter affiliation, and cognitive attributes, so that the gold-standard metadata applied to the processed items without alteration.

### Test materials

The test material was a 60-item comprehensive endocrinology examination developed in accordance with the Chinese National Medical Licensing Examination blueprint. It spanned eight content chapters and four item types: single-choice (*n* = 48), multiple-choice (*n* = 6), short-answer (*n* = 5), and essay (*n* = 1).

For each item, eight metadata fields were extracted: question number, question type, score, difficulty, chapter affiliation, cognitive level (awareness/familiarity/mastery, per Chinese medical-education standards), cognitive objective (recall/understanding/application, based on a modified Bloom’s taxonomy), and knowledge points. The gold-standard key for all 480 field values was prepared independently by two experienced medical educators, with discrepancies resolved by discussion to full agreement.

### AI model selection

Twelve LLMs were evaluated, selected on the basis of public availability as of May 2026, Chinese-language support, the ability to process medical text, and the ability to follow structured task instructions. Eight were domestic models: Doubao (ByteDance), DeepSeek-V4.0 (DeepSeek), ERNIE 5.1 (Baidu), GLM-5.1 (Zhipu AI), Kimi K2.6 (Moonshot AI), MedSeek V1.0 (a medical-education LLM jointly developed by the Peking University Health Science Center and the Chaoxing Digital-Intelligence Joint Laboratory), Hunyuan 3.0 (Tencent), and Qwen 3.7 (Alibaba). Four were international models: Claude Sonnet 4.6 (Anthropic), ChatGPT GPT-5.5 (OpenAI), Gemini 3.5 Flash (Google), and Grok 4.0 (xAI). The version identifiers above correspond to the versions accessed during the study window.

All models were accessed through their official web interfaces using free-tier or standard subscription accounts under default parameters. No system prompts, temperature settings, or other generation parameters were modified. This deliberately reflects the conditions a typical user would encounter, rather than an optimized API configuration; the consequences of this ecologically valid setup for reliability are considered in the Results and Discussion.

We restricted the evaluation to general-purpose conversational LLMs that could be accessed through an official, Chinese-accessible web interface on a free or standard account and could accept an uploaded document together with structured task instructions. Models built for specialized modalities or narrow domains, such as video or image generation, or single-purpose vertical applications, were outside the scope of this study. On these criteria, several widely known models were not included. Meta’s Llama models and Huawei’s Pangu were excluded because no web-based access meeting these conditions was available to us at the time of the study. Quark was excluded as redundant, since it is built on the same Qwen foundation as the already-included Qwen 3.7. iFLYTEK Spark was accessed but failed to complete the structured task in a usable form, its output collapsed structurally partway through the paper, and was therefore not carried into the comparative analysis. These criteria were applied uniformly across all candidate models.

### Task protocol

A single standardized prompt was used for all models and all runs. The prompt provided the examination as an uploaded WORD document and instructed the model to extract the eight metadata fields for all 60 items and return them as a table with a fixed column order (question number, question type, score, cognitive level, cognitive objective, chapter, knowledge points, difficulty). To accommodate platform differences, the prompt specified that if a model could not generate a downloadable Excel file, it should instead present all 60 items as a table within the conversation. A result was counted as delivered whether it was exported as a file or returned as an in-conversation table, provided the structured field data could be retrieved intact; the delivery format did not affect scoring, as only the extracted field values were compared against the key. The complete prompt is provided as a Supplementary appendix.

Each model performed this identical task in three independent runs. Runs were conducted in separate sessions to ensure independence, and all runs for all models were completed within the 25 May–3 June 2026 window (during revision) to minimize confounding from model updates. Each run was carried out by the same operator under identical conditions, who recorded the end-to-end wall-clock time with a stopwatch, measured from submission of the prompt to completion of the output. When a model returned fewer than the full 60 items, refused, or failed to produce any usable output, the run was recorded as such and retained in the analysis rather than being repeated or discarded.

### Outcome measures and scoring

Rather than collapse multiple dimensions into a single weighted composite, we used three complementary metrics. Completion rate (items attempted/60, averaged over three runs) indexes whether a model reliably engages with the full task. Conditional accuracy (proportion of items with all eight fields correct, over completed runs only) indexes capability when the model responds. Task Success Rate (TSR), the primary metric, is the number of items with all eight fields simultaneously correct, divided by 60 and averaged over three runs, with failed runs scored as zero; by penalizing refusal and error alike, it reflects whether output can be deployed without per-item correction. Reporting all three keeps reliability, capability, and deployable performance distinct rather than conflated.

A field was scored correct only when it matched the gold-standard key, after a fixed set of normalization rules was applied identically to all models: full and half-width spaces were collapsed, Chinese and ASCII commas treated as equivalent, question type matched on its core term, chapter names compared ignoring leading section numbers, and scores compared as numbers after removing the unit character “分.” These rules reconciled surface form only; semantic content fields were never normalized, so a genuine misjudgment could not be masked. Runs producing no output (refusal, timeout, or system cut-off) were scored zero and retained in the denominator, and out-of-order responses were matched to the correct key item by question type, chapter, and knowledge point before scoring. End-to-end run time was additionally recorded by stopwatch and is reported as a corroborating signal of reliability rather than a primary outcome.

### Statistical analysis

Data are reported as mean ± standard deviation (SD); the SD is interpreted substantively, as a direct index of cross-run reliability rather than merely as a measure of estimation uncertainty. Two pre-specified comparisons used standard nonparametric tests, chosen because the metrics were bounded percentages with non-normal, high-variance distributions across a small number of models. To assess whether capability and deployability diverged, conditional accuracy and TSR were compared across the twelve models using the Wilcoxon signed-rank test, with the two values paired within each model. To assess whether model origin was associated with performance, domestic and international models were compared on TSR using the Mann–Whitney U test. Values of *p* < 0.05 are considered significant, and all analyses were performed in Python 3.10.

## Results

### Overall task success

We evaluated twelve LLM on a single 60-item endocrinology examination, extracting eight metadata fields per item across three independent runs per model. We adopt TSR as the primary metric: an item counts as successful only if all eight fields are simultaneously correct, averaged across the three runs with failed runs scored as zero. TSR therefore penalizes refusal and error alike, reflecting deployability rather than peak capability. We additionally report two component layers, including completion rate and conditional accuracy, so that reliability and capability can be read separately. Table 1 reports all three, ranked by TSR.

**Table 1:** Overall performance of twelve models, ranked by task success rate (mean ± SD across three runs).

Rank	Model	Group	TSR (%)	Compl. (%)	Cond. acc. (%)	Dominant failure mode
1	Claude Sonnet 4.6	Intl	100 ± 0	100 ± 0	100	—
2	Doubao	Dom	99.4 ± 1.0	100 ± 0	99.4	—
2	DeepSeek-V4	Dom	99.4 ± 1.0	100 ± 0	99.4	—
4	Grok 4.0	Intl	98.9 ± 1.0	100 ± 0	98.9	Single-line format collapse
5	ERNIE 5.1	Dom	93.3 ± 8.7	100 ± 0	93.3	Column misalignment (1 run)
6	GLM-5.1	Dom	66.7 ± 57.7	66.7 ± 57.7	100	File-parsing refusal (1 run)
6	Kimi K2.6	Dom	66.7 ± 57.7	66.7 ± 57.7	100	Tool-round exhaustion (1 run)
8	Gemini 3.5 Flash	Intl	33.3 ± 57.7	33.3 ± 57.7	100	Capability refusal (2 runs)
9	MedSeek V1.0	Dom	32.8 ± 56.8	33.3 ± 57.7	98.3	Timeout/silent failure (2 runs)
10	ChatGPT GPT-5.5	Intl	25.6 ± 8.4	100 ± 0	25.6	Knowledge-point truncation
11	Hunyuan 3.0	Dom	22.8 ± 22.4	37.8 ± 36.5	60.1	Dropped columns/reordering
12	Qwen 3.7	Dom	6.1 ± 1.9	9.4 ± 1.0	66.7	Example trap (5/60 items)

TSR, all-eight-fields-correct items/60, averaged over three runs, failed runs scored 0. Completion, items attempted/60. Conditional accuracy, all-fields-correct items/items attempted, within completed runs only. Dom, domestic; Intl, international.

Performance was sharply bimodal: five models reached a TSR of 90% or higher and the remaining seven fell below 70%, with none in between (median 66.7%, range 6.1–100%, IQR 68.0). This reflects a clean split between models that are deployable for this task and those that are not, rather than a performance continuum. The lower tier was heterogeneous in cause: some models stayed accurate when they completed but failed entirely on one or more runs (GLM-5.1, Kimi K2.6), others completed every run yet were systematically inaccurate (ChatGPT GPT-5.5), and one delivered only 5 of 60 items on every attempt (Qwen 3.7). The standard deviations are as telling as the means, with values near ±57.7, indicating that a model’s three runs included both a success and a total failure, a pattern examined in the next section.

Group origin did not predict performance: domestic and international models did not differ significantly in TSR (median 66.7% vs. 66.1%; Mann–Whitney U = 13.0, *p* = 0.67), and both groups contained both top performers and failure-prone models. Reliability differences here were a property of individual models, not of national origin.

### Reliability: cross-run variance as the decisive factor

A model that extracts metadata correctly when it succeeds is of little practical value if it fails unpredictably. To separate capability from reliability, we compared each model’s conditional accuracy (correctness when a run completes) against its TSR (correctness across all three runs, failures scored as zero). Because the two metrics are paired within each model, we tested their difference with the Wilcoxon signed-rank test.

Conditional accuracy was significantly higher than TSR across the twelve models (median 99.2% vs. 66.7%; Wilcoxon signed-rank, *p* = 0.016). The gap was driven by six models, all showing reductions of 33 percentage points or more (Table 2). Critically, GLM-5.1, Kimi K2.6, and Gemini 3.5 Flash each achieved a conditional accuracy of 100%. On this metric alone, their performance was indistinguishable from the top-ranked models. However, one or two out of their three test runs failed entirely, causing their TSR to drop to 66.7% or below. A single-run evaluation, which by construction observes only conditional accuracy, would have ranked these high-variance models among the best performers.

**Table 2:** Conditional accuracy versus TSR, illustrating the reliability gap (ranked by gap).

Model	Cond. Acc. (%)	TSR (%)	Gap (pp)	Profile
Gemini 3.5 flash	100	33.3	66.7	High accuracy, unreliable
MedSeek V1.0	98.3	32.8	65.5	High accuracy, unreliable
Qwen 3.7	66.7	6.1	60.6	Partial delivery every run
Hunyuan 3.0	60.1	22.8	37.3	Unstable output structure
GLM-5.1	100	66.7	33.3	High accuracy, unreliable
Kimi K2.6	100	66.7	33.3	High accuracy, unreliable
ChatGPT GPT-5.5	25.6	25.6	0	Stable but low quality
ERNIE 5.1	93.3	93.3	0	Stable, high quality
Grok 4	98.9	98.9	0	Stable, high quality
DeepSeek-V4	99.4	99.4	0	Stable, high quality
Doubao	99.4	99.4	0	Stable, high quality
Claude sonnet 4.6	100	100	0	Stable, high quality

Gap, conditional accuracy − TSR (percentage points). A large gap indicates that capability is undermined by run-to-run failure. A zero gap is not by itself a marker of quality: ChatGPT GPT-5.5 is stable yet uniformly low, whereas Claude and DeepSeek are stable and high.

### Failure modes: behavioural versus platform-level

To address whether these failures reflect remediable prompting problems or intrinsic limitations, we classified every failed or degraded run by its proximate cause. Twelve failures emerged across two categories with very different implications for deployment (Table 3). Behavioural failures arise from a model’s own output strategy or self-assessment and may be partially mitigated by prompting. Common cases include the example trap, in which a model delivers only the first few items as if the task were complete (Qwen 3.7 returned 5 of 60 items on every run), and capability refusal, in which a model declines as “only a language model” (Gemini, MedSeek V1.0). Platform-level failures are imposed by the serving infrastructure and cannot be removed by prompting: Kimi K2.6 was cut off by tool-round exhaustion, and MedSeek V1.0 failed silently after an inference timeout, returning nothing after one to 2 minutes.

**Table 3:** Failure modes, by category and remediability.

Category	Failure mode	Model(s)	Removable by prompting?
Behavioural	Example trap (delivers only 5–6 items)	Qwen 3.7, ChatGPT GPT-5.5	Partial: intrinsic output strategy
Behavioural	Capability refusal (“only a language model”)	Gemini, MedSeek V1.0	Difficult: guardrail/self-concept
Behavioural	Knowledge-point truncation/rewriting	ChatGPT GPT-5.5	Partial: semantic compression
Behavioural	Single-line format collapse	Grok 4	Yes: output formatting
Behavioural	Column misalignment (level ↔ objective)	ERNIE 5.1	Partial
Behavioural	Dropped column /reordering/collapse	Hunyuan 3.0	Partial to none
Platform	File-parsing refusal (declines.docx)	GLM-5.1	Partial: change file format
Platform	Tool-round exhaustion (system cut-off)	Kimi K2.6	No: platform/framework limit
Platform	Silent timeout (~1–2 min, no output)	MedSeek V1.0	No: inference timeout

“Removable by prompting” reflects whether better instruction design could plausibly eliminate the failure. Platform-level failures recurred under identical prompts and are determined by the serving environment, not the request.

This distinction is the direct answer to the concern that such failures are merely prompting artefacts. Some behavioural failures might be reduced with better instructions, but the platform-level failures lie outside the user’s control and recurred under identical prompts; they would persist in any realistic deployment. Reliability, in this setting, is a system-level property, not solely a function of prompt quality.

### Field-level accuracy: a structural-to-semantic gradient

Aggregating across all completed runs (approximately 1,140 item-instances per field), accuracy varied systematically by field type, following a clear structural-to-semantic gradient (Table 4). Purely structural fields were extracted almost perfectly (score value and question type: 100%) and categorical fields followed closely (difficulty 98.8%, chapter 98.7%). The semantic fields were harder: cognitive objective (98.7%) and cognitive level (98.1%) required interpreting the item’s intent, and knowledge point, the most open-ended field, was the clear floor at 88.2%.

**Table 4:** Field-level accuracy across all completed runs, by field category.

Field	Category	Accuracy (%)
Score value	Structural	100
Question type	Categorical	100
Difficulty	Categorical	98.8
Chapter	Categorical	98.7
Cognitive objective	Semantic	98.7
Cognitive level	Semantic	98.1
Knowledge point	Semantic	88.2

Accuracy pooled over all completed runs; failed runs are excluded here (they are captured by TSR and completion rate).

A methodological caveat underlies these figures. How a field is scored, particularly whether trivial formatting differences are reconciled before comparison, can change a model’s apparent accuracy dramatically. Doubao illustrates this most starkly. Its extracted content was almost entirely correct, but it used different surface conventions from the answer key: extra spaces, hyphen spacing, and “2 型” where the key wrote “2型.” Compared character-for-character, these cosmetic mismatches count as errors and Doubao’s accuracy appears to be only 72.8%; once formatting is normalised it rises to 99.4%, representing a shift of more than 26 percentage points from a single scoring decision.

Crucially, normalisation reconciles surface form only, never content. We applied a fixed set of rules identically to every model: full-width and half-width spaces were collapsed; Chinese and ASCII commas were treated as equivalent; question type was matched on its core term (“single-choice,” “multiple-choice,” etc.) ignoring enumerative prefixes such as “一、”; chapter names were compared ignoring any leading section number; and score values were compared as numbers after removing the unit character “分.” The semantic content fields, including cognitive level, cognitive objective, knowledge point and difficulty, were never collapsed: “了解” (aware) and “掌握” (master) remained distinct, so a genuine misjudgement could never be normalised away. Because these rules touch only orthography and are applied symmetrically across all models, they correct a systematic bias against models whose formatting differs from the key without rewarding any model for substantive error. The full rule set is specified in Methods.

### Normative override: models overruling the author’s judgement

One finding in the cognitive-level field was qualitatively distinct and, for educational use, the most consequential. Item 1, an introductory overview question, had been assigned the level “aware” (了解) by the author, fitting its place at the start of the curriculum. Four models (ERNIE 5.1, Doubao, MedSeek V1.0 and DeepSeek V4.0) instead reassigned it to “master” (掌握), the highest level. They did not fail to read the label; they replaced it with a judgement of their own.

This was not random error. All four deviations ran in the same direction, upgrading a foundational item, and the pattern cut across vendors rather than reflecting one model’s quirk (DeepSeek V4.0 was mildest, overriding in only one of three runs). We term this normative override (or silent relabeling): rather than extracting the label the author assigned, the model substitutes its own judgement and quietly overwrites the human annotation. Because cognitive-level labels govern item-difficulty distribution and paper assembly, a tool that silently rewrites an author’s pedagogical decisions cannot be trusted to build an item bank, however accurate it is elsewhere. Tellingly, this failure is invisible to aggregate accuracy and surfaces only under item-level scrutiny.

### Run time as a corroborating signal of reliability

We recorded each run’s end-to-end time with a stopwatch under the same web-interface conditions. These times are not a controlled speed benchmark, since they include network and platform latency and rest on three runs per model, but they corroborate the reliability picture from an independent angle (Table 5).

**Table 5:** Run time (mm:ss) and outcome across three runs.

Model	Run 1	Run 2	Run 3
DeepSeek V4.0	0:29 60 items	0:28 60 items	0:29 60 items
Doubao	0:48 60 items	0:38 60 items	0:42 60 items
Grok 4	0:59 60 items	0:28 60 items	0:35 60 items
ChatGPT GPT-5.5	0:55 60 items	0:56 60 items	0:52 60 items
ERNIE 5.1	1:05 60 items	1:07 60 items	1:11 60 items
Claude Sonnet 4.6	1:25 60 items	1:27 60 items	1:20 60 items
Qwen 3.7	1:10 5 items	0:49 5 items	0:42 5 items
Hunyuan 3.0	1:48 10 items	1:58 10 items	2:28 48 items
GLM-5.1	2:38 60 items	2:00 failed (file refusal)	3:05 60 items
Kimi K2.6	2:55 60 items	4:01 60 items	6:00 failed (no output)
MedSeek V1.0	1:00 failed (no output)	1:46 60 items	2:00 failed (no output)
Gemini 3.5 Flash	0:46 failed (refusal)	0:17 failed (refusal)	0:58 60 items

Stopwatch-measured end-to-end durations under default web-interface settings. “items” = items returned in that run; failed runs are marked. Completion percentages in Table 1 derive from full scoring, whereas the counts here reflect items returned on screen. Reported as a corroborating signal, not a controlled speed benchmark.

Failure typically took the form of prolonged hanging followed by no output, not a fast error: MedSeek V1.0 returned nothing after one to 2 minutes, and Kimi K2.6 ran a full 6 minutes before being cut off empty-handed. Reliable models showed the opposite signature: DeepSeek V4.0, Doubao, and Grok 4 returned all sixty items in under a minute, tightly clustered across their three runs. A third pattern, fast refusal, appears in Gemini’s sub-minute declines. In this setting, the duration of a run signaled less how fast a model worked than whether it would deliver at all.

In sum, conditional accuracy was uniformly high, but it did not determine deployability: the decisive differences lay in cross-run variance, not mean accuracy, and the failures responsible were often system-level rather than prompting artefacts. National origin was not a meaningful predictor; reliability was a property of individual models. For this class of tools, the question is not which model is most accurate, but which can be trusted to deliver, completely and faithfully, every time.

## Discussion

This study evaluated large language models as assessment-automation tools, rather than as test-takers answering questions. Across twelve models and three independent runs each, the central finding is not which model was most accurate, but that accuracy alone was a poor guide to whether a model could be deployed. Most models, when they completed the task, were highly accurate; what separated usable from unusable tools was cross-run reliability. Several models that looked excellent on any single run failed outright on another, and these failures, rather than differences in mean accuracy, were what determined practical suitability. We therefore treat model origin as largely irrelevant to performance and reposition run-to-run reliability as the decisive criterion for this class of educational tasks.

A single evaluation run is insufficient to characterize these tools. Several models in our sample were flawless whenever they completed the task yet failed entirely on one or more runs, so that a conditional accuracy near 100% coexisted with a far lower task success rate. Observed only once, as in a conventional benchmark, such models would be ranked among the best and their unreliability would go undetected. This concern is not specific to our task: a recent re-evaluation of state-of-the-art models across three runs found single-run leaderboards highly unstable, inverting at least one pairwise ranking in 83% of cases relative to a three-run majority (Gonzalez et al., 2025). The practical implication is that variance, not the mean, is what matters when an institution must decide whether a tool can be trusted in routine use, and that reporting cross-run variance is a direct measure of deployability rather than a statistical formality.

If reliability hinges on whether a model fails, the nature of those failures determines whether they can be engineered away. Classifying every failed run by its proximate cause revealed two categories with sharply different implications. Behavioural failures, such as an output strategy that delivers only the first few items or a refusal framed as “I am only a language model,” originate in the model itself and might be reduced through more elaborate prompting, although prompt design has well-documented limits even for tasks within a model’s competence (Vilakati, 2025). Platform-level failures are different in kind: Kimi K2.6 was cut off by tool-round exhaustion and MedSeek v1.0 stopped silently after an inference timeout; and both recurred under identical prompts. These arise from the serving infrastructure, not the instruction, and no amount of prompt refinement would remove them. This distinction matters because it answers a natural objection, namely that such failures merely reflect inadequate prompting. Some do; but the platform-level failures are properties of how a model is deployed and served, and would persist for any user in any realistic setting. Reliability, in other words, is a system-level property rather than a question of instruction quality alone (Huan and Wang, 2026; Magnus, 2026).

These reliability differences did not align with model origin. Domestic and international models were statistically indistinguishable on task success, and each group contained both the most dependable performers and outright failures: the single flawless model was international, but two domestic models matched it almost exactly, while failures appeared on both sides. This pattern is consistent with a growing body of work showing that Chinese-trained models now rival or exceed their international counterparts on Chinese medical tasks (Yao et al., 2025; Xiong et al., 2025; Hajjo et al., 2026; Sallam et al., 2025). Our contribution is to add a cautionary qualification to that literature: parity on accuracy does not imply parity on reliability, and neither dimension tracks national origin in any systematic way. For an institution choosing a tool, the relevant question is not whether a model is domestic or international, but whether a specific model behaves dependably on the specific task at hand.

A subtler risk emerged beyond outright failure. As reported above, four models independently upgraded an introductory item from the cognitive level the author had assigned to the highest level, a systematic, same-direction deviation rather than random error. We term this silent relabeling: the model does not misread the label but quietly replaces it with a judgement of its own. This is more damaging than a simple extraction error, because cognitive-level labels govern difficulty distribution and paper assembly; a tool that silently rewrites pedagogical decisions cannot be trusted to populate an item bank, however accurate it is elsewhere. The finding reinforces a recurring conclusion that LLMs require human oversight for judgement-laden educational tasks (Bolgova et al., 2025) and supports a division of labour in which models handle extraction while educators retain authority over normative decisions (Rareș et al., 2025).

This division of labour is also visible in the field-level results. Accuracy followed a clear structural-to-semantic gradient: numeric and categorical fields such as score and question type were extracted almost perfectly, whereas the open-ended knowledge-point field was the hardest. This suggests a practical workflow in which structural fields are automated with minimal review while semantic fields, especially knowledge points and cognitive level, receive targeted human verification, concentrating expert effort where models are weakest and least trustworthy (Li et al., 2026). Such a tiered approach aligns extraction capability with the level of oversight each field warrants, rather than treating the task as uniformly automatable.

If such a workflow is to be useful beyond the present study, its findings must extend past a single endocrinology examination at one institution. We expect the core distinction, that cross-run reliability rather than accuracy governs deployability, to hold broadly, because its causes are not discipline-specific: the failure modes we observed, including truncated delivery, capability refusal, tool-round exhaustion, and inference timeouts, stem from how models generate and how platforms serve them, not from the medical content of the items. The underlying task, extracting tagged metadata from a document into a structured table, is likewise not unique to medicine; it recurs in item-bank construction and test assembly across disciplines. The framework we propose, namely repeated runs, reporting of cross-run variance, and a human-in-the-loop division of labour, should therefore apply to this broader class of structured extraction tasks. This expectation is consistent with large-scale evidence that LLM capability varies substantially across exams, languages, and contexts, so that no single accuracy figure transfers cleanly to a new setting (Zong et al., 2024). What changes across settings is likely to be the particulars rather than the principle: absolute accuracy and failure rates will differ with discipline, item format, and language, and the structural-to-semantic gradient may shift for tasks dominated by free-text or case-based items. Validating the approach at scale will be aided by emerging benchmarks that pair questions with rich metadata annotations (Zong et al., 2026), which could allow reliability to be assessed across many specialties at once. The concern is not confined to educational metadata: in clinical feature extraction, frameworks that explicitly penalize overconfident errors have sharply reduced hallucinated and missing fields (Abumelha et al., 2025), indicating that task-specific reliability, rather than raw model capability, is the operative issue across structured medical extraction tasks, and that targeted methods can improve it.

### Limitations and future directions

First, this study used a single 60-item examination in one discipline; as discussed above, the core distinction between accuracy and reliability is expected to generalize, but the specific accuracy and failure rates require confirmation in other settings. Second, three runs per model are enough to show that a model can fail, a fact single-run designs miss, but not enough to estimate failure probability precisely; and the normative-override finding, though consistent across four models, rests on a single item and warrants confirmation on more. Third, because models were accessed through public web interfaces under default settings, the results reflect real deployment conditions but are not fully controlled and represent a snapshot of rapidly evolving systems. Future work should extend this reliability-centered approach across disciplines and item types, use more runs to estimate failure probability directly, and move the field toward reporting standards that treat cross-run variance, rather than single-run accuracy, as a primary outcome.

## Conclusion

For automated extraction of assessment metadata, LLMs are capable but unevenly reliable, and it is reliability, not accuracy, that determines whether a model can be deployed. Several models that were accurate when they succeeded failed unpredictably across runs, a pattern invisible to single-run evaluation and unrelated to national origin. Such tools should therefore be assessed by repeated runs that report cross-run variance, and used within a tiered workflow that automates structural fields while reserving human judgement for semantic ones.

## Data Availability

The original contributions presented in the study are included in the article/Supplementary material, further inquiries can be directed to the corresponding author.
