# Supplementary material for: Beyond accuracy: evaluating the reliability of large language models for medical assessment
Source: Front Artif Intell. 2026 Jul 8;9:1832829. doi: 10.3389/frai.2026.1832829 (PMC13388558; doi:10.3389/frai.2026.1832829)
Supplement: Supplementary file 1 [file Data_Sheet_1.DOCX]

**Standardized Task Prompt**

The following prompt was used identically for all models and all runs. The Chinese version is the original prompt as administered; an English translation is provided for reference.

**Original prompt** (Chinese, as administered):

请分析我上传的 Word 文档，这是一份考试试题，共 60 个题目。请逐题提取以下 8 项信息，填入 Excel 表格。如果平台不支持生成可下载的 Excel 文件，请在对话中以表格形式显示全部 60 题，确保每一题都有完整的 8 个字段。

提取字段：

1. 题号
2. 题型（一、单选题 / 二、多选题 / 三、简答题 / 四、论述题）
3. 分值
4. 认知水平（从标签提取：掌握 / 熟悉 / 了解）
5. 认知目标（从标签提取：记忆 / 理解 / 综合应用）
6. 章节（从标签提取：一内分泌系统总论 / 二内分泌器官的形态结构 / 三下丘脑-垂 体相关疾病 / 四甲状腺相关疾病 / 五肾上腺相关疾病 / 六糖代谢异常 / 七脂代谢异常 / 八钙-磷代谢）
7. 知识点（从"知识点："字段提取，多个知识点用中文逗号分隔）
8. 难度（从"难度："字段提取：易 / 中 / 难）

输出格式（表头固定顺序）：

题号 | 题型 | 分值 | 认知水平 | 认知目标 | 章节 | 知识点 | 难度

**English translation** (for reference):

Please analyze the Word document I have uploaded; it is an examination containing 60 items. For each item, extract the following 8 fields and enter them into an Excel table. If the platform cannot generate a downloadable Excel file, present all 60 items as a table within the conversation, ensuring every item has all 8 fields.

Fields to extract:

1. Item number
2. Item type (I. single-choice / II. multiple-choice / III. short-answer / IV. essay)
3. Score
4. Cognitive level (extract from the label: mastery / familiarity / awareness)
5. Cognitive objective (extract from the label: recall / understanding / application)
6. Chapter (extract from the label: 1. Endocrine System Overview / 2. Endocrine Organ Structure / 3. Hypothalamic–Pituitary Diseases / 4. Thyroid Diseases / 5. Adrenal Diseases / 6. Glucose Metabolism Disorders / 7. Lipid Metabolism Disorders / 8. Calcium–Phosphorus Metabolism)
7. Knowledge points (extract from the "knowledge points:" field; separate multiple points with a Chinese comma)
8. Difficulty (extract from the "difficulty:" field: easy / moderate / hard)

Required output format (fixed column order):

Item number | Item type | Score | Cognitive level | Cognitive objective | Chapter | Knowledge points | Difficulty
